# Lyme Carditis: An Interesting Trip to Third-Degree Heart Block and Back

**DOI:** 10.1155/2016/5454160

**Published:** 2016-11-06

**Authors:** Maxwell Eyram Afari, Fady Marmoush, Mobeen Ur Rehman, Umama Gorsi, Joseph F. Yammine

**Affiliations:** Department of Medicine, Memorial Hospital of Rhode Island, Alpert Medical School, Brown University, Pawtucket, RI, USA

## Abstract

Carditis is an uncommon presentation of the early disseminated phase of Lyme disease. We present the case of a young female who presented with erythema migrans and was found to have first-degree heart block which progressed to complete heart block within hours. After receiving ceftriaxone, there was complete resolution of the heart block in sequential fashion. Our case illustrates the importance of early recognition and anticipation of progressive cardiac conduction abnormalities in patients presenting with Lyme disease.

## 1. Introduction

Lyme disease is the most common tick-borne disease in the United States and Europe. Lyme disease is transmitted by the* Ixodes* tick (*scapularis* and* pacificus* in North America,* persulcatus *in Asia, and* ricinus* in Europe). The disease is mostly commonly caused by* Borrelia burgdorferi *in North America and by* Borrelia afzelii, Borrelia burgdorferi, and Borrelia garinii* in Europe and Asia. Lyme disease can evolve into a multisystemic disease and its clinical presentation can vary widely. The clinical spectrum of Lyme disease is classified into (1) early localized phase (days to weeks after the tick bite, presenting with erythema migrans and a nonspecific viral syndrome), (2) early disseminated phase (weeks to months after the tick bite, presenting with cardiac and/or neurologic manifestations), and (3) late phase (months to years after the erythema migrans, accompanied by arthritis and/or neurological symptoms such as headache and lymphocytic meningitis).

## 2. Case

We present the case of a thirty-three-year-old woman with no significant past medical history, who presented to the Emergency Department during the summer reporting a three-day history of intermittent, dull, moderate-intensity chest discomfort. Review of systems was remarkable for shortness of breath and lightheadedness. Three weeks earlier, she had presented to the same Emergency Department complaining of photophobia, headache, and fever (101.4°F). A lumbar puncture at that time was unremarkable. Travel history was remarkable for hiking in the mountains of New Hampshire a month prior to presentation.

**Figure 1:**
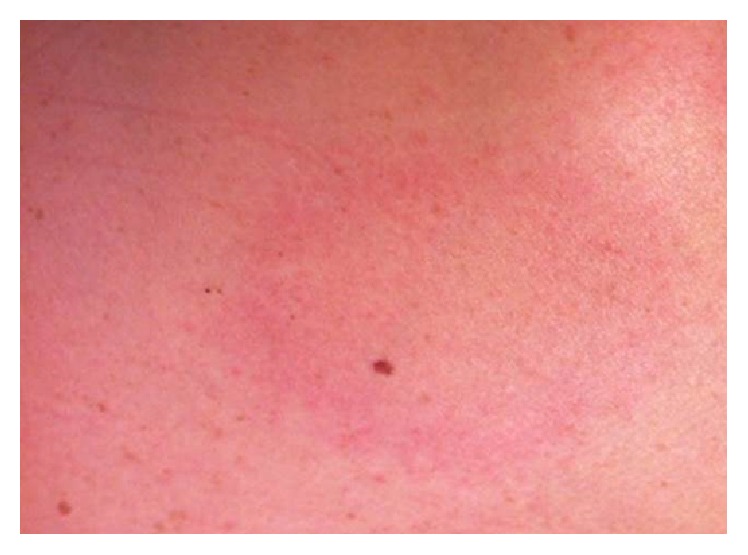
Five cm erythematous macular rash with central clearing on the posterior aspect of the patient's neck.

**Figure 2:**
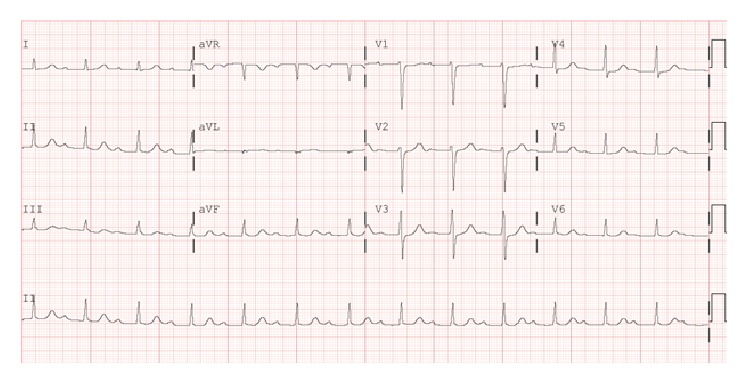
Electrocardiogram showing first-degree atrioventricular block with PR interval of 320 ms.

**Figure 3:**
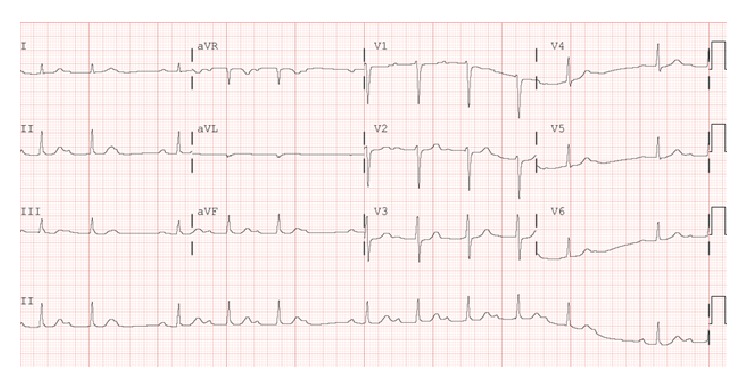
Electrocardiogram showing Mobitz Type 1 AV block.

**Figure 4:**
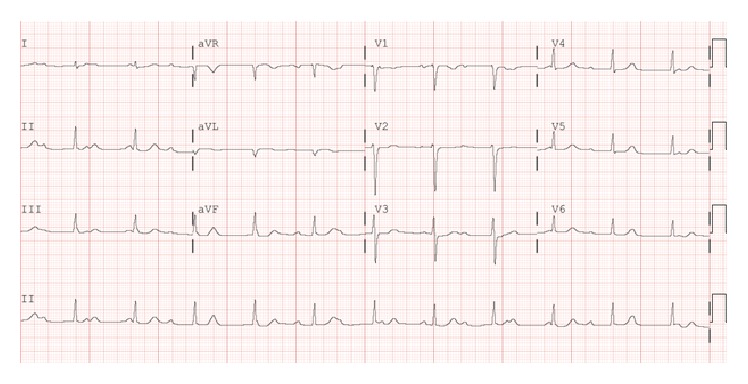
Electrocardiogram showing third-degree heart block.

Initial physical evaluation revealed blood pressure of 112/68 mmHg, heart rate of 86 beats per minute, respiratory rate of 15 breaths per minute, and temperature of 99.0°F. Cardiopulmonary examination was unremarkable. The skin exam revealed a 5 cm erythematous macular rash with central clearing on the posterior aspect of the neck (Figure 1). Chest radiograph and laboratory tests, including cardiac enzymes (Troponin I: 0.01 (reference < 0.04)), were unrevealing. An initial electrocardiogram showed a first-degree AV block with PR interval of 320 milliseconds (Figure 2). A presumptive diagnosis of early disseminated phase of Lyme disease was made, and the patient was admitted to the cardiac telemetry floor. Several hours after admission, telemetry monitoring revealed second-degree (Mobitz Type 1) AV block as shown in Figure 3. Shortly thereafter, complete heart block was noted (Figure 4), with no additional clinical or hemodynamic changes. Simultaneously it was noted that due to a sign-out error, the patient had not received the first dose of ceftriaxone, which was then promptly administered.

After receiving three doses of ceftriaxone, the repeat EKG showed regression of the complete heart block back to Mobitz Type 1 AV block and then first-degree AV block [i.e., exact reverse sequence]. Her echocardiogram revealed no structural abnormalities. She was diagnosed with Lyme disease based on the presence of erythema migrans and* Borrelia* antibodies demonstrated by enzyme-linked immunosorbent assay and western immunoblot findings (IgG bands: 28, 30, 39, 41, 45, and 58 and IgM: 39 and 41). Serology testing for Babesiosis and Ehrlichiosis, concomitant infections occasionally reported with acute Lyme disease, was negative. The patient was discharged home four days after admission to complete a 21-day course of once daily intravenous ceftriaxone therapy. One month later when the patient was seen in an outpatient cardiology clinic for follow-up, she was asymptomatic and her electrocardiogram showed a normal sinus rhythm.

## 3. Discussion

Lyme carditis is an uncommon presentation of early disseminated Lyme disease and occurs during the early disseminated phase of this condition. Steere et al. were the first to publish a case series on this clinical entity in 1980 [1]. Although Lyme carditis can manifest as myocarditis, pericarditis, and/or left ventricular dysfunction, its most common presentation is an electrical conduction disorder [1]. The most common symptoms of Lyme carditis include chest pain, palpitation, dizziness, and shortness of breath. Rarely, syncope and sudden cardiac death have also been reported. In the case series by Steere et al., PR interval >300 milliseconds was associated with progression of AV block [1]. In Lyme carditis, the conduction abnormality is commonly supra-Hisian, typically in the AV node. There have been rare reports of an infra-Hisian involvement indicated by QRS widening. Some other rare conduction abnormalities reported include QT prolongation, alternating bundle branch block, and ventricular and supraventricular tachyarrhythmias.

By far the most common conduction disorder involves the atrioventricular (AV) node, causing various AV blocks from first degree to complete heart block. In a review of over 100 cases, van der Linde reported that approximately 12% of cases presented with first-degree AV block, 16% as second-degree AV block, and 49% as third-degree heart block [2]. The rest of the cohort (23%) did not have any conduction abnormalities. The worsening of the degree of AV block may occur within minutes as demonstrated in our case. In the review by van der Linde, 35% of the cohort had required temporary pacing, and only 5.7% required a permanent pacemaker of which only one patient remained pacer dependent. Lyme carditis is a reversible disorder and in the vast majority of cases a permanent pacemaker is not required. In a systematic review of Lyme carditis associated third-degree heart block permanent pacemaker was required in only two (4.4%) out of forty-five cases [3].

It is believed that, at the early disseminated stage of Lyme disease, the spirochete spreads hematogenously to the targeted organ systems. Spirochetes have been isolated in the heart tissue from patients with myocarditis and pancarditis at autopsy [4]. It is suspected that the immunological inflammatory response to the spirochete in the cardiac layer and electrical system explains the AV conduction disorders. Animal models have gone a long way to elucidate the pathophysiology of Lyme carditis. Monocyte and macrophages are the main inflammatory cells in Lyme carditis [5]. They increase the expression of the chemoattractant protein 1 RNA which appears to increase the severity of Lyme carditis [6]. In mice, infection is shown to upregulate the proinflammatory cytokines such as interleukin 1*β* and TNF-*α*. They are detected as early as 3 days after infection, with TNF-*γ* persisting for at least 42 days [7]. Experimentally it has also been shown that, through immunologic mimicry, IgM antibodies against the spirochetes may cross-react with heart tissues causing inflammatory response [8]. A previous report demonstrated a late gadolinium enhancement consistent with focal myocarditis in the atrioventricular region of a patient with Lyme carditis [9].

Our case highlights how rapidly the conduction disorder in Lyme carditis can fluctuate; thus it is very important that patients carrying this diagnosis are admitted to the telemetry unit. Lyme carditis can be self-limited; however, antibiotics are required to shorten the disease course and minimize cardiovascular complications. Mild Lyme carditis (first-degree AV block, PR interval < 300 ms) can be treated with doxycycline or amoxicillin, while advanced conduction abnormalities (high grade AV block, PR > 300 mSec) as diagnosed in our patient necessitate treatment with ceftriaxone or Penicillin G. The choice of ceftriaxone in our patient was due to her presentation with active symptoms along with advanced heart block.

## 4. Conclusion

Our case highlights the importance of considering Lyme disease as an etiology of acute AV nodal conduction disorders in patients who present with cardiac symptoms. This case highlights the importance of taking a good travel history and emphasizes the importance of appropriate and timely therapy to prevent unnecessary interventions such as permanent pacemaker insertion.
